# Spatial and temporal dynamic changes of oral microbiome in removable partial denture wearers: a longitudinal study using full-length 16S rRNA sequencing

**DOI:** 10.1080/20002297.2025.2589655

**Published:** 2025-11-26

**Authors:** Xin Feng, Xueqi Gan, Biao Ren, Ziqianhong Wan, Yuxuan Wang, Xuanyi Gao, Zhuoli Zhu

**Affiliations:** a State Key Laboratory of Oral Diseases & National Center for Stomatology & National Clinical Research Center for Oral Diseases & Department of Dental Technology, West China Hospital of Stomatology, Sichuan University, Chengdu, Sichuan, China; b State Key Laboratory of Oral Diseases & National Center for Stomatology & National Clinical Research Center for Oral Diseases & Department of Prosthodontics, West China Hospital of Stomatology, Sichuan University, Chengdu, Sichuan, China; c State Key Laboratory of Oral Diseases & National Center for Stomatology & National Clinical Research Center for Oral Diseases, West China Hospital of Stomatology, Sichuan University, Chengdu, Sichuan, China; d State Key Laboratory of Oral Diseases & National Center for Stomatology & National Clinical Research Center for Oral Diseases & Department of Geriatric Dentistry, West China Hospital of Stomatology, Sichuan University, Chengdu, Sichuan, China

**Keywords:** Removable partial dentures, oral microbiota, older adults, 16S rRNA, dental plaque

## Abstract

**Objective:**

This longitudinal study aimed to characterize the spatial and temporal dynamics of oral microbiome colonization on removable partial dentures (RPDs) and corresponding dental surfaces at species-level resolution, to elucidate ecological succession patterns and identify potential pathogenic colonizers.

**Methods:**

We conducted a longitudinal study of 10 participants requiring RPDs. Plaque samples were collected from four sites at five time points. The microbial communities were profiled using PacBio full-length 16S rRNA sequencing, enabling high accuracy taxonomic assignment to the species level. Bioinformatic analyses included alpha/beta diversity, LEfSe, and PICRUSt2 functional prediction.

**Results:**

Significant differences in microbial composition were observed between RPD and dental plaques, despite similar alpha diversity. Temporal analysis revealed a progressive decrease in RPD plaque diversity. Notably, the potential respiratory pathogen *Klebsiella pneumoniae* was detected in early RPD biofilms. A three-stage ecological succession model for RPD biofilm was proposed, initiating with acidogenic pioneers, followed by functional amplification of taxa involved in extracellular polysaccharide production, and culminating in a stable, acid-tolerant community.

**Conclusion:**

This study provides a species-level understanding of microbiome changes associated with RPDs, confirms differences between RPD plaque and dental plaque, proposes a succession model for RPD-associated bacteria, and determines key turning points and potential pathogens.

## Introduction

With the acceleration of global aging, oral health problems among older adults have become a critical public health concern [1]. The removable partial denture (RPD) serves as an essential therapeutic device for restoring masticatory function and esthetics in older adults with partial edentulism [2]. However, prolonged use of RPDs inevitably changes the oral microenvironment by creating stagnation zones that shield microbial deposits from salivary clearance and mechanical hygiene practices [3]. These sheltered niches promote persistent microbial colonization through intimate contact with mucosal and dental surfaces [4]. Notably, age-related decreases in metabolic activity and self-cleaning capacity increase susceptibility to pathogenic biofilm accumulation [5]. Studies indicate that RPD plaque is not only a direct cause of oral diseases, such as denture stomatitis and oral malodor [6,7], but also can induce microbial dysbiosis and harbor opportunistic pathogens, which may lead to pneumonia through the aspiration of pathogens or to conditions such as bacterial endocarditis, posing a serious threat to the systemic health of older adults [8,9]. An increased risk of pneumonia has been observed among RPD wearers, particularly those who wear RPDs overnight or maintain poor denture hygiene [10–12]. In contrast, good daily cleaning and wearing habits of dentures may partially reduce the risk of pneumonia [13]. In addition, RPD use may also increase the risk of cardiovascular disease and diabetes mellitus [14–16].

Despite preliminary studies of RPD-associated microbial communities exist. Early research mainly relied on traditional isolation and culture techniques and focused more on specific opportunistic pathogens, such as *Candida albicans* [17–19]. Although some studies have used 16S rRNA sequencing to reveal relationships between RPD and natural tooth plaque, their limited resolution prevents the precise identification of disease-relevant species [19,20]. Shi et al. investigated the oral microbiota associated with RPDs in healthy individuals and those experiencing stomatitis. They reported considerable microbial similarity between the biofilms colonizing RPD surfaces and the natural teeth within the same individual, with higher phylogenetic similarity than comparisons across different subjects. This pattern indicates a host-dependent microbial profile [20]. Paradoxically, another study performing spatial analyses revealed distinct ecological divergence between denture communities and adjacent tooth plaques despite microenvironmental contiguity [19]. Existing longitudinal studies on RPD-associated microbiota have focused primarily on *C. albicans* or explored changes only at the phylum level, without microbiota succession of RPD and teeth [21]. One study investigated the impact of RPDs on periodontal disease at the species level [22]. However, no study has systematically mapped the spatial and temporal succession patterns of RPD-associated microbiota at species-level resolution.

The 16S rRNA gene sequencing is a widely used method for the taxonomic profiling of bacterial communities [23,24]. However, conventional next-generation sequencing (NGS) platforms are constrained by short-read limitations. This partial gene coverage severely compromises species-level discrimination, particularly among closely related taxa [25]. In contrast, third-generation sequencing (TGS) technologies enable full-length amplification of the V1–V9 regions through long-read sequencing and have achieved species-level exploration [26]. These technologies minimize the PCR amplification biases inherent in NGS workflows while achieving superior coverage depth [27]. Despite these advantages, TGS applications in oral microbiome research remain scarce [28–30].

In summary, previous studies have relied on short-read sequencing, limiting species-level exploration. Moreover, no study has simultaneously tracked multisite biofilm succession at the interface between RPDs and natural teeth. Thus, this study applied TGS by PacBio and combined it with a longitudinal sampling design to achieve species-level resolution and perform high-precision microbiome analyses of natural dental and RPD plaques at different sites. By analyzing the spatial specificity and temporal dynamics of bacterial structures, we aim to track the succession of bacterial communities over time, identify key species and predict their functional characteristics, thereby providing an evidence base for early warning and precise intervention in RPD-related oral diseases.

## Materials and methods

### Sample size

The required sample size for this study was estimated using G*Power software version 3.1.9.7, based on differences in alpha diversity (Shannon index) reported in prior research. The parameters for this calculation comprised an effect size (Cohen's *d*) of 1.12, which was derived from the mean Shannon index difference between RPD and dental plaque [19], 80% statistical power, and a significance threshold of 0.05. Consequently, the analysis required at least eight participants for paired *t*-testing; however, 10 subjects were enrolled to accommodate an anticipated dropout rate of approximately 25%.

### Sample collection

The study was approved by the Ethics Committee of West China Hospital of Stomatology Sichuan University (WCHSIRB-D-2022-038). Ten participants with partial edentulism were recruited from the Department of Geriatric Dentistry, West China Hospital of Stomatology, Sichuan University. Informed written consent was obtained from all study participants. The participants were assessed by experienced physicians, and suitable RPDs made of a metal framework with acrylic resin bases were designed. The exclusion criteria are listed in Appendix Table 1. All participants underwent supragingival and subgingival scaling one week before RPD placement to reset the oral microbiota to a baseline level across all participants and minimize the influence of pre-existing, long-standing plaque. The one-week interval allowed for sufficient recovery of salivary flow and the gingival crevicular environment while preventing the re-establishment of highly complex and stable microbial ecosystems typical of long-standing plaque [31–33]. After scaling, dental plaques were sampled at five time points: 7 days before RPD placement (t_7), immediately after placement (t0), and at 1 day (t1), 7 days (t7) and 30 days (t30) after placement. RPD plaques were sampled at t1, t7 and t30. Samples were collected from four sites: the proximal contact area of the RPD (DP), the corresponding tooth surface (TP), the RPD retaining clasp (DC) and the corresponding tooth contact surface (TC). Each site was sampled separately at all time points. During the sampling period, the same management of participants was adopted. The participants were required to wear RPDs for at least 2 h before sampling and avoid eating, drinking or cleaning teeth and RPDs during this period. Plaque samples were collected by dentists using flocked swabs in a circular motion. All dental plaques were originated from natural teeth. During the study period, the participants were instructed to wear RPDs for at least 8 h per day. The natural teeth and RPDs were brushed twice daily with a standardized soft brush and toothpaste, and the RPDs were soaked overnight in a standardized denture cleaner. The collected plaque specimens were individually transferred to 1.5 mL microcentrifuge tubes and promptly frozen at −80 °C for subsequent DNA isolation.

### DNA extraction and PCR amplification

Genomic DNA was extracted from the samples utilized the FastDNA SPIN Kit designed specifically for soil samples (MJYH, Shanghai, China). The integrity of the extracted DNA was evaluated via electrophoresis on a 1% agarose gel, and the DNA purity and concentration were measured using a NanoDrop 2000 spectrophotometer (Thermo Scientific, Wilmington, USA). DNA samples were considered qualified if gel electrophoresis showed clear bands at the correct positions and a concentration above 0.5 ng/μL. The amplification of bacterial 16S rRNA genes was conducted with the universal primers 27F (5’-AGRGTTYGATYMTGGCTCAG-3’) and 1492 R (5’-RGYTACCTTGTTACGACTT-3’) [34], modified to include barcodes compatible with PacBio sequencing for multiplex analyses. The polymerase chain reaction (PCR) mixtures totaled 20 μL, comprising 10 μL of 2 × Pro Taq, 0.8  μL of each primer (5  μM), 10 ng of template DNA, and nuclease-free water up to 20 μL. Amplification involved an initial denaturation at 95 °C for 3 min, followed by 29 cycles at 95 °C for 30 s, annealing at 60 °C for 30 s, and extension at 72 °C for 45 s, with a final extension at 72 °C for 10 min. PCR amplifications were replicated thrice using an ABI GeneAmp® 9700 thermal cycler. The PCR amplicons were subsequently purified with AMPure® PB beads (Pacific Biosciences, CA, USA) and quantified utilizing Synergy HTX (Biotek, USA).

### DNA library construction and sequencing

Equimolar concentrations of the purified PCR products were combined, and the DNA sequencing library was prepared with the SMRTbell prep kit 3.0 (Pacific Biosciences, CA, USA). The prepared SMRTbell libraries were sequenced on the PacBio Sequel IIe System (Pacific Biosciences, CA, USA), was performed by Majorbio Bio-Pharm Technology Co., Ltd. (Shanghai, China).

### Data processing

Raw sequence data obtained from PacBio sequencing were processed using SMRT Link analysis software (version 11.0). Circular consensus sequencing (CCS) reads were separated based on barcode sequences and filtered according to length criteria (1,000–1,800 bp) to ensure full-length amplification of the 16S rRNA gene. Denoising of HiFi reads employed DADA2 [35] plugin integrated within the QIIME2 [36] software package (version 2020.2), applying standard recommended settings to achieve single-nucleotide resolution by profiling sample-specific error patterns. The resulting denoised reads, termed amplicon sequence variants (ASVs), were rarefied to the minimum sequence number per sample, maintaining an average Good’s coverage above 80%. The Ribosomal Database Project (RDP) classifier (v2.13), embedded in QIIME2, was utilized with the NT_16S database (v20230830) to perform taxonomic assignments of ASVs. Metagenomic functional prediction was conducted using Phylogenetic Investigation of Communities by Reconstruction of Unobserved States (PICRUSt2) [37].

### Statistical analysis

Bioinformatic analysis was conducted using the Majorbio Cloud platform. Based on the ASV data, alpha diversity indices, including observed ASVs and the Shannon index were calculated using Mothur v1.30.2 [38]. Principal coordinate analysis (PCoA) using weighted UniFrac distances was performed with the Vegan (v2.4.3) package to assess microbial community similarity. PERMANOVA was utilized to determine the proportion and significance of variance attributed to the experimental conditions. Additionally, the Kruskal‒Wallis test facilitated comparative analysis of predicted Kyoto Encyclopedia of Genes and Genomes (KEGG) Orthology (KO) functions between distinct groups.

## Results

### Spatial comparison

**Figure 1.**
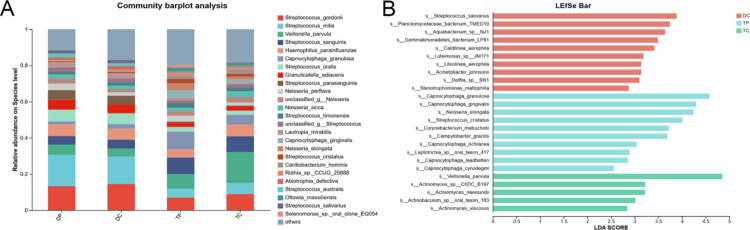
Relative abundance of bacterial species (A) and LEfSe analysis (B) at the proximal contact area of RPD (DP), corresponding tooth surface (TP), RPD retaining clasp (DC), and corresponding tooth contact surface (TC). A logarithmic LDA score threshold of 2 was set to identify discriminative features.

A total of 160 samples were grouped according to collection sites (TC, TP, DC, DP) to determine the relative bacterial abundance at the species level for spatial comparisons (Figure 1A). The bacterial species accounting for more than 1% of the total microbiota at each sampling location are detailed in the Appendix Table 2; species constituting less than 1% of the total microbiota were grouped collectively under ‘others’. The analysis showed notable variations in microbial composition between the removable RPD surfaces and the dental plaque. Although *Streptococcus* consistently emerged as the predominant genus across all the samples, the distribution and abundance of specific species differed depending on the sampling site. In RPD plaque, *Streptococcus mitis*, *Streptococcus gordonii* and *Streptococcus oralis* were more abundant, along with *Haemophilus parainfluenzae, Veillonella parvula* and *Granulicatella adiacens*. In dental plaque, the dominant species was *V. parvula*, followed by *S. sanguinis* and *S. gordonii*. *V. parvula* showed significantly higher abundance in TC compared to TP, while *C. granulosa* dominated in TP but decreased in abundance in TC.

Microbial communities were compared among the four groups at the species level using LEfSe analysis (Figure 1B). There were 10, 10 and 5 taxa enriched in the DC, TP and TC groups, respectively (LDA score > 2). In the DC group, pathogenic bacteria such as *Stenotrophomonas maltophilia* showed significant enrichment. In the TP group, *Capnocytophaga gingivalis* and *Capnocytophaga granulosa*, which are associated with periodontal diseases, were significantly abundant. In the TC group, *V. parvula* exhibited the greatest enrichment.

**Figure 2.**
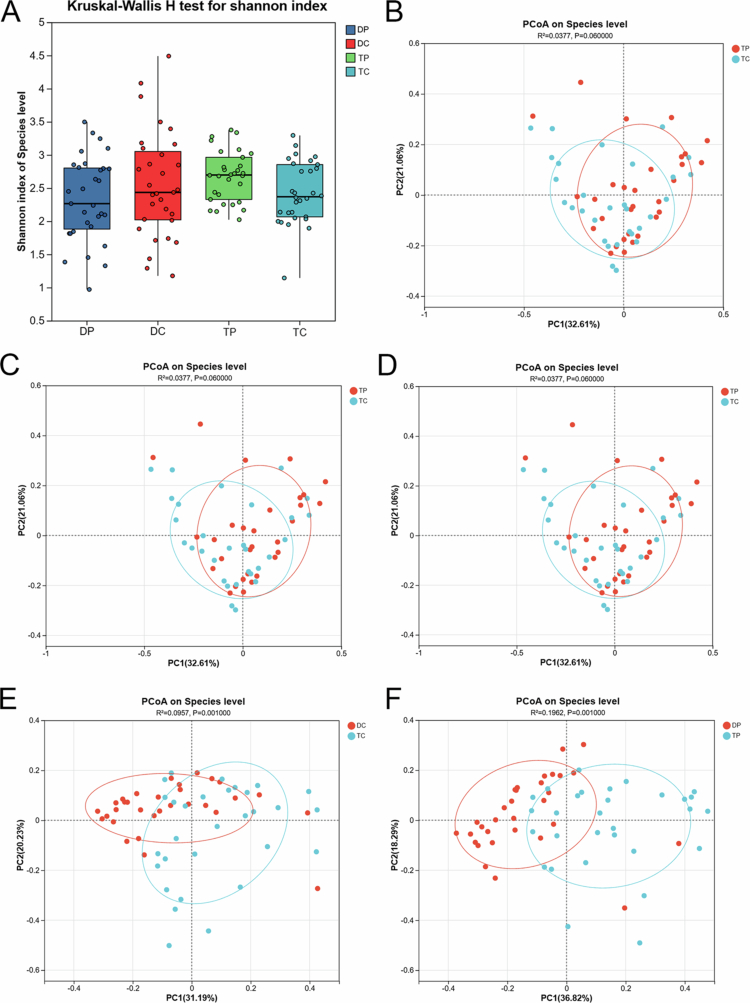
Comparative analysis of bacterial diversity between dental plaque and removable partial denture (RPD) surfaces at distinct sites (DP, TP, DC, TC) was performed. (A) Assessment of alpha diversity using the Shannon index showed no statistically significant variations in microbial richness or evenness among the groups (*p* > 0.05). Each sample group is represented by a distinct color coding. (B–F) PCoA based on weighted UniFrac distances illustrating beta diversity. Statistical significance was evaluated with PERMANOVA.

Alpha diversity analysis (Shannon index) showed no significant difference in microbial evenness or richness among the four groups (*P* > 0.05, Figure 2A). Beta diversity analysis (PCoA) confirmed significant microbial composition differences among the sampling sites (*P* < 0.05). Specifically, the microbial communities from different sites were similar (Figure 2B). Similarly, the microbial communities at different RPD plaque sites did not differ significantly (*P* > 0.05, Figure 2C). However, there were significant differences between dental and RPD plaque microbial communities (Figure 2D‒F).

### Temporal comparison

The samples were grouped into dental groups (t_7, t0t, t1t, t7t, t30t) and RPD groups (t1d, t7d, t30d) according to the sampling times for temporal comparisons. The relative bacterial abundance at the species level was determined. Species exceeding 1% relative abundance per sampling site are listed in the Appendix Tables 3 and 4.

**Figure 3.**
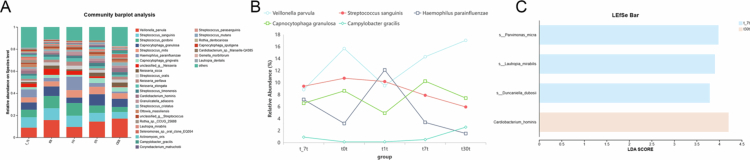
Analysis of bacterial species relative abundance (A), successional changes of dominant species (B) and linear discriminant analysis effect size (LEfSe) (C) in dental plaque samples collected at various time intervals: 7 days prior to RPD insertion (t_7t), immediately following insertion (t0t), and subsequently at 1 day (t1t), 7 days (t7t) and 30 days (t30t) postinsertion. Discriminative taxa were identified using a logarithmic LDA score cutoff of 2.

Dental plaque microbial communities exhibited dynamic changes (Figure 3A). At t_7, the community was dominated by *S. sanguinis* and *V. parvula*, exhibiting high diversity. At t0t, *V. parvula* increased dramatically to 15.69%, while *S. gordonii* and *S. sanguinis* dominated. By t1t, *H. parainfluenzae* emerged as a new dominant species. From t7t to t30t, the microbial community gradually stabilized, *V. parvula* regained dominance. *Campylobacter gracilis* significantly increased, and *Streptococcus* maintained a high abundance (Figure 3B).

Microbial communities at the species level of dental plaque between the five dental plaque groups using LEfSe analysis were compared (Figure 3C). Only two groups have a significant abundance of taxa (LDA score > 2). In t30t group, *Cardiobacterium hominis* had the most significant abundance.

**Figure 4.**
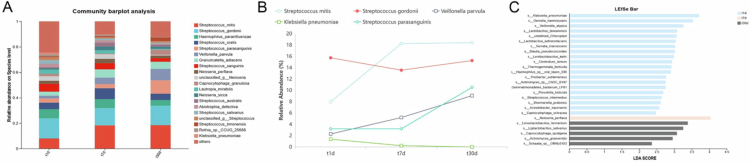
Assessment of bacterial species relative abundance (A), successional changes in dominant species (B) and LEfSe analysis (C) in plaque obtained from RPD surfaces at 1 day (t1d), 7 days (t7d) and 30 days (t30d) postplacement. A logarithmic LDA score cutoff of 2 was applied to detect discriminative taxa.

The RPD plaque communities also showed temporal dynamics (Figure 4A). At t1d, *S. gordonii* dominated, while *H. parainfluenzae* and *S. mitis* had a high abundance. By t7d, *S. mitis* replaced *S. gordonii* as the dominant species. At this stage, *S. oralis* and *G. adiacens* were enriched. At t30d, the microbial communities stabilized, with *S. mitis* codominating alongside *S. gordonii.* Additionally, *S. parasanguinis* and *V. parvula* significantly increased. Notably, the potential pathogen *Klebsiella pneumoniae* appeared at t1d, but its abundance sharply decreased with prolonged RPD wearing. The proportion of the ‘others’ category decreased from 24.67% to 12.36% (Figure 4B).

LEfSe analysis revealed significantly enriched species-level taxa in RPD plaques among the three groups (Figure 4C). The t1d, t7d and t30d groups showed enrichment of 20, 1 and 5 taxa, respectively (LDA score > 2). At t1d, pathogenic bacteria (*K. pneumoniae* and *Acinetobacter baumannii*) associated with pneumonia showed significant abundance. *Limosilactobacillus fermentum* and *Ligilactobacillus salivarius* had the most significant abundance in the t30d group.

**Figure 5.**
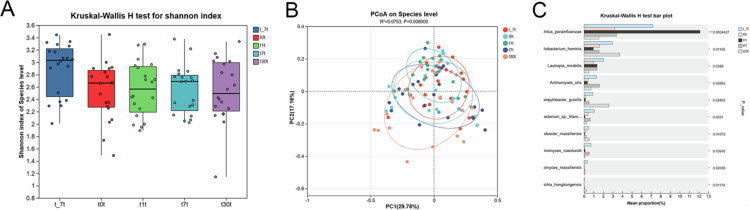
Diversity of bacterial communities in dental plaque samples at multiple intervals: 7 days pre-RPD insertion (t_-7t), at insertion time (t0t), and at 1 day (t1t), 7 days (t7t) and 30 days (t30t) afterward. (A) Shannon index-based alpha diversity analyses indicated no significant temporal shifts in microbial richness or evenness (*p* > 0.05). (B) Weighted UniFrac-based beta diversity evaluated through PCoA. Statistical significance was determined by PERMANOVA (*p* < 0.05). (C) Multigroup comparative analysis identifying taxa with significantly varied abundances.

**Figure 6.**
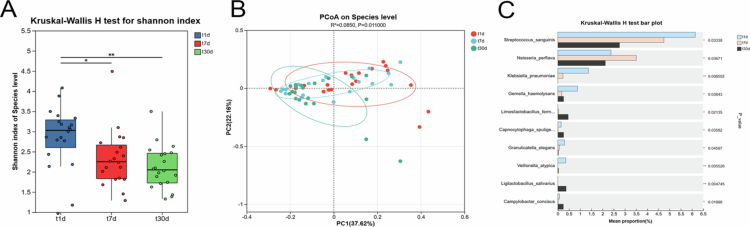
Diversity analysis of RPD plaque bacterial communities collected at 1 day (t1d), 7 days (t7d) and 30 days (t30d) after RPD placement. (A) Alpha diversity (Shannon index) analysis showed the microbial evenness and richness decreased over time (*p* > 0.05). (B) Beta diversity (PCoA) plot based on weighted UniFrac distances. *P*-values were calculated using PERMANOVA (*p* < 0.05). (C) Multiple-group comparisons highlighting significant differences in taxa abundance.

The Shannon index, employed to assess alpha diversity, indicated no statistically significant shifts in microbial richness or evenness within the dental plaque communities over the evaluated period (Figure 5A). Conversely, alpha diversity indices for microbiota colonizing RPD surfaces exhibited a marked reduction over the observation timeframe (Figure 6A). Beta diversity, assessed using PCoA, further confirmed significant temporal differences in microbial community structure between the dental plaque and RPD surfaces (Figures 5B and 6B).

Multiple comparisons showed significant changes in the abundance of landmark bacterial species (*P* < 0.05) on the dental and RPD surfaces over time. On tooth surfaces, *H. parainfluenzae* and *C*. h*ominis* abundances changed significantly (Figure 5C). On RPD surfaces, *S. sanguinis*, *Neisseria perflava*, *K. pneumoniae* and *Gemella haemolysans* abundances significantly changed over time (Figure 6C).

**Figure 7.**
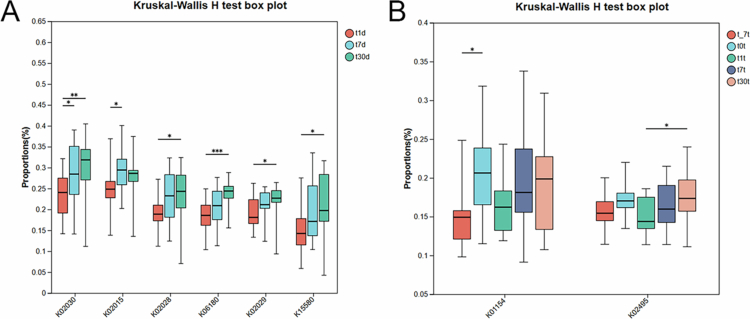
Predicted functional differences among microbial communities. (A) Identification of the top 20 significantly differentially expressed KO genes in RPD plaque collected at 1 day (t1d), 7 days (t7d), and 30 days (t30d) postplacement. The genes included the following: K02030 (polar amino acid transport substrate-binding protein); K02015 (iron complex permease protein); K02028 (ATP-binding protein of the polar amino acid transport system); K06180 (23S rRNA pseudouridine1911/1915/1917 synthase); K02029 (polar amino acid permease protein); K15580 (substrate-binding protein of the oligopeptide transport system). (B) Top 20 KO genes with significant differences in expression abundance in dental plaque collected at 7 days before RPD placement (t_-7t), immediately after placement (t0t), and at 1 day (t1t), 7 days (t7t) and 30 days (t30t) after RPD placement. K01154, type I restriction enzyme, S subunit; K02495, oxygen-independent coproporphyrinogen III oxidase.

PICRUSt2 functional predictions indicated significant increases over time in KO genes related to amino acid transport systems, iron transport, and rRNA modification enzymes on RPD surfaces (Figure 7A). Few KO genes differed significantly in the dental groups over time (Figure 7B).

## Discussion

This study used TGS by PacBio combined with a longitudinal sampling design to examine spatial and temporal changes in the dental and RPD-associated microbial communities. Compared to NGS, TGS enhances species-level resolution and reduces PCR amplification bias, improving taxonomic quantification accuracy [26,27]. This approach allowed more precise identification of the bacterial composition on RPD and tooth surfaces at various time points and sampling sites compared to previous studies using next-generation sequencing (NGS). The results showed that no significant difference in microbial community evenness or richness between dental and RPD plaque. Beta diversity analysis revealed significant differences in microbial species composition between these two plaque types. However, there were no significant differences among the dental plaque sites, and a similar pattern was observed among the RPD plaque sites. This finding may result from differences in surface properties between RPDs and natural enamel, causing preferential colonization by specific bacteria [39]. Additionally, although RPD plaque composition is partially influenced by dental plaque [19], other studies have demonstrated that RPD hygiene significantly influences bacterial complexity in plaque communities [40–42]. In this study, unified oral and denture hygiene protocols may have reduced the microbial complexity on RPD surfaces. Supragingival and subgingival scaling performed one week before RPD placement removed mature dental plaque, ensuring that the observed microbial dynamics reflected the impact of RPD rather than preexisting plaque [31]. This approach also allowed sufficient recovery of the salivary microbiota and gingival crevicular fluid flow [32,33]. Given that microbial communities across various periodontal conditions are most comparable within the 3–7-day interval following scaling [31], sampling in this investigation was strategically performed on the 7 day post-scaling period to elucidate the influence of RPD placement on the establishment of microbial populations.

When grouped by sampling site, *Streptococcus* was the most dominant genus in all groups, which is consistent with observations by Fujinami et al. [19]. However, this study explored species-level differences and revealed that *S. mitis*, *S. gordonii* and *S. oralis* were more abundant in RPD plaque, whereas *V. parvula* was more abundant in dental plaque*.* Notably, the early dominance of *Streptococci*, such as *S. gordonii*, is critical for initial biofilm formation, whose surface proteins provide adhesion and aggregation sites for other oral bacteria. Meanwhile, the LEfSe results revealed that in the DC group, *S. maltophilia* was identified in significant abundance. This emerging global opportunistic pathogen is associated not only with stomatitis but also with respiratory infections, which are of particular concern for immunocompromised individuals [43,44]. Its ability to colonize RPDs positions the denture as a potential reservoir for respiratory pathogens, highlighting the importance of denture hygiene. This difference may be due to the frequent wearing of RPDs, which create aerobic conditions that affect microbial colonization and succession. Natural dental surfaces subjected to saliva erosion and mechanical cleaning likely favor anaerobic bacteria [19]. Additionally, the elevated presence of *V. parvula* in the TC group likely reflects its adaptation to the anaerobic microenvironment beneath RPD contact surfaces [45], which is also a well-established niche for biofilm formation [46]. In contrast, the proximity of the TP group to the gingival crevices may increase *C. granulosa* through nutrient-rich crevicular fluid [47,48].

This study divided the samples into RPD and dental plaque groups based on time points. The alpha diversity of the dental plaque microbial communities showed no significant change over time, while that of RPD plaque gradually decreased. This trend may result from improved oral hygiene and resource competition or metabolic inhibition of dominant bacteria [39,49]. Beta diversity analysis showed significant temporal differences in microbial composition between dental and RPD plaques, revealing dynamic microbial changes. The baseline period of dental plaque was dominated by *S. sanguinis* and *V. parvula*. After 7 days, *V. parvula* significantly increased, although *S. gordonii* and *S. sanguinis* remained dominant. Facultative anaerobic bacteria such as *H. parainfluenzae* rapidly became dominant at t1, but decreased in abundance. In the long term, bacterial populations gradually returned to stability, and *V. parvula* regained dominance. The significant increase of *C. gracilis* after long-term RPD wearing warrants attention, as its sulfur metabolites may damage tissue and increase periodontitis risk [22]. Additionally, LEfSe analysis showed a significant presence of *C. hominis* at t30t, associated with endocarditis [50].

To characterize microbial dynamics systematically, a three-stage ecological succession model of RPD biofilm was proposed. In the early colonization stage, acidogenic pioneer species (*S. gordonii* and *H. parainfluenzae*) initiate biofilm formation by glycolysis, generating lactic acid. This acidic microenvironment selects for acid-tolerant species, such as *V. parvula*, which further metabolize lactate into propionate. This process resembles the early demineralization phase of dental caries [45]. During the intermediate phase (functional bacterial amplification), *S. mitis* produces a biofilm matrix using extracellular polysaccharide synthesis (EPS). Concurrently, enrichment of *G. adiacens* indicates complex metabolic networks. This EPS-driven structural maturation parallels cariogenic biofilm development, where glucans increase bacterial cohesion and acid retention [51]. In the final phase, acid-resistant bacteria further acidify the microenvironment, creating a dysbiotic similar to caries progression, selecting for acid-resistant bacteria [45]. LEfSe analysis revealed that *L. fermentum* was significantly abundant at t30d, which is related to good EPS production [52]. Moreover, the increased abundance of *V. parvula* may produce propionate, which, at high concentrations, induces hepatic gluconeogenesis [53]. The abundance of a lactate-utilizing bacterium signifies a key metabolic interaction within the biofilm, where it consumes organic acids produced, potentially influencing the local pH [54].

PICRUSt2 predictions preliminarily validated this model, which closely parallels caries pathogenesis [45,51]. The polar amino acid transport system (K02030, K02028, K02029) supports EPS synthesis through efficient nitrogen source uptake, providing a structural foundation for biofilm formation [55]. Iron-complex transport system permease proteins may temporarily help opportunistic pathogens (*K. pneumoniae*) occupy niches, though their abundance subsequently decreases, possibly due to host immune responses or hygiene interventions [56]. Additionally, this transport system has potential for Candida infections through siderophore production [57]. Increased oligopeptide transport system substrate-binding proteins may aid dominant bacteria in simplifying community structure through nitrogen-source competition, facilitating biofilm transition from loose to dense forms [58]. Oxygen-independent coproporphyrinogen III oxidase associated with iron and heme acquisition in *Porphyromonas gingivalis* coincides with increased *C. gracilis*, suggesting an increased risk of periodontitis [22,59]. However, this model remains a hypothesis derived from correlation-based analyses. While PICRUSt2 predictions provide valuable functional context, they are inferred from 16S data rather than direct metagenomic or metabolomic measurements, may overlook the metabolic characteristics of uncultured bacteria [37]. Therefore, metabolomic validation is crucial to confirm the predicted acidification and metabolic interactions, which would significantly strengthen the robustness of this model.

Wong et al. used metagenomic analysis and reported that respiratory pathogens such as *Streptococcus pneumoniae* and *Pseudomonas aeruginosa* were significantly enriched in the saliva of RPD wearers [14]. Another study reported that RPDs might serve as reservoirs for respiratory pathogens, including *S. pneumoniae, P. aeruginosa* and *Moraxella catarrhalis* [17]. Additionally, *Streptococcus, Haemophilus*, *Neisseria*, *Prevotella* and *Veillonella* are the dominant bacterial genera associated with healthcare-related pneumonia and are relatively high abundance on RPD surfaces [19,60]. Most of these bacterial genera were identified on RPD surfaces in the present study. LEfSe analysis showed the significant enrichment of *K. pneumoniae* and *A. baumannii* in the t1d group, which are closely associated with pneumonia development. Although the abundance of *K. pneumoniae* significantly decreased with prolonged wearing, these findings underscore the importance of intensive denture hygiene during the early wearing phase. A synergistic cleaning strategy that combines ultrasonic cleaning with effective chemical agents such as 0.12% chlorhexidine digluconate or NitrAdine has shown a strong antibacterial effect [42,61].

This study had a small sample size and used a cross-sectional design. Despite strict exclusion criteria and unified participant management, the cohort needs to be expanded and the observation period should be extended to reveal the long-term effects of RPD wearing. Future studies should integrate multiomics technologies to comprehensively characterize the impact of RPDs on oral microecology and validate the proposed three-stage successional model. Additionally, fungal communities were not assessed. Given the clinical relevance of Candida spp. in RPD-related stomatitis, future research should incorporate ITS sequencing to investigate cross-kingdom interactions and identify keystone pathogens.

In conclusion, this study explores the effect of RPD on the spatial and temporal dynamics of the oral microbiota associated with RPDs using TGS and a longitudinal sampling design. A three-stage successional model of RPD-associated bacterial biofilms was proposed, and several pathogens associated with RPD and dental plaque were identified. The findings provide new insights into oral microbiome shifts following RPD placement and offer a scientific basis for developing early intervention strategies for RPD wearers.

## Supplementary Material

Supplementary material Supplementary tables.

## Data Availability

All raw data of the sample has been uploaded to NCBI (SRP590784).

## References

[1] Lozada-Martinez ID, Diazgranados-Garcia MC, Castelblanco-Toro S, et al. Global research on centenarians: a historical and comprehensive bibliometric analysis from 1887 to 2023. Ann Geriatr Med Res. 2024;28(2):144–155. doi: 10.4235/agmr.24.0043 38584431 PMC11217658

[2] Friel T, Waia S. Removable partial dentures for older adults. Prim Dent J. 2020;9(3):34–39. doi: 10.1177/2050168420943435 32940586

[3] Marsh PD. Dental plaque as a microbial biofilm. Caries Res. 2004;38(3):204–211. doi: 10.1159/000077756 15153690

[4] Sang T, Ye Z, Fischer NG, et al. Physical-chemical interactions between dental materials surface, salivary pellicle and streptococcus gordonii. Colloids Surf B Biointerfaces. 2020;190:110938. doi: 10.1016/j.colsurfb.2020.110938 32172164 PMC7440396

[5] Ikebe K, Gondo Y, Kamide K, et al. Occlusal force is correlated with cognitive function directly as well as indirectly via food intake in community-dwelling older Japanese: from the SONIC study. PLoS One. 2018;13(1):e0190741. doi: 10.1371/journal.pone.0190741 29304177 PMC5755890

[6] McReynolds DE, Moorthy A, Moneley JO, et al. Denture stomatitis-an interdisciplinary clinical review. J Prosthodont. 2023;32(7):560–570. doi: 10.1111/jopr.13687 36988151

[7] Wyszyńska M, Nitsze-Wierzba M, Białożyt-Bujak E, et al. The problem of halitosis in prosthetic dentistry, and new approaches to its treatment: a literature review. JCM. 2021;10(23):5560. doi: 10.3390/jcm10235560 34884262 PMC8658399

[8] Lim TW, Li KJKW, Wang J, et al. Association between removable denture-wearing and systemic diseases: a scoping review. J Dent. 2025;161:105977. doi: 10.1016/j.jdent.2025.105977 40669603

[9] Dakka A, Nazir Z, Shamim H, et al. Ill effects and complications associated to removable dentures with improper use and poor oral hygiene: a systematic review. Cureus. 2022;14(8):e28144. doi: 10.7759/cureus.28144 36148203 PMC9482451

[10] Alzamil H, Wu TT, van Wijngaarden E, et al. Removable denture wearing as a risk predictor for pneumonia incidence and time to event in older adults. JDR Clin Trans Res. 2021;8(1):23800844211049406–75. doi: 10.1177/23800844211049406 34693793 PMC9772962

[11] Kusama T, Aida J, Yamamoto T, et al. Infrequent denture cleaning increased the risk of pneumonia among community-dwelling older adults: a population-based cross-sectional study. Sci Rep. 2019;9(1):13734. doi: 10.1038/s41598-019-50129-9 31551442 PMC6760190

[12] Twigg JA, Smith A, Haury C, et al. Compositional shifts within the denture-associated bacteriome in pneumonia – an analytical cross-sectional study. J Med Microbiol. 2023;72(6). doi: 10.1099/jmm.0.001702 37341468

[13] Takeuchi K, Izumi M, Furuta M, et al. Denture wearing moderates the association between aspiration risk and incident pneumonia in older nursing home residents: a prospective cohort study. Int J Environ Res Public Health. 2019;16(4):554. doi: 10.3390/ijerph16040554 30769876 PMC6406796

[14] Liu Y, Qin H, Li T, et al. Denture use and risk for cardiometabolic disease: observational and mendelian randomization analyses. Eur J Prev Cardiol. 2024;31(1):13–20. doi: 10.1093/eurjpc/zwad295 37697428 PMC10767255

[15] Liang X, Chou OHI, Cheung BMY. The association between denture use and cardiovascular diseases. The United States National Health and nutrition examination survey 2009–2018. Front Cardiovasc Med 2022. 2023;9:1000478. doi: 10.3389/fcvm.2022.1000478 PMC9871755 36704477

[16] Lee JH, Han JS, Han K, et al. Association between Diabetes and the use of removable dental prostheses among the korean population. J Korean Med Sci. 2019;34(41):e262. doi: 10.3346/jkms.2019.34.e262 31650717 PMC6813424

[17] O’Donnell LE, Robertson D, Nile CJ, et al. The oral microbiome of denture wearers is influenced by levels of natural dentition. PLoS One. 2015;10(9):e0137717. doi: 10.1371/journal.pone.0137717 26368937 PMC4569385

[18] O’Donnell LE, Smith K, Williams C, et al. Dentures are a reservoir for respiratory pathogens. J Prosthodont. 2016;25(2):99–104. doi: 10.1111/jopr.12342 26260391

[19] Fujinami W, Nishikawa K, Ozawa S, et al. Correlation between the relative abundance of oral bacteria and *Candida albicans* in denture and dental plaques. J Oral Biosci. 2021;63(2):175–183. doi: 10.1016/j.job.2021.02.003 33662564

[20] Shi B, Wu T, McLean J, et al. The denture-associated oral microbiome in health and stomatitis. mSphere. 2016;1(6):e00215-16. doi: 10.1128/mSphere.00215-16 28066812 PMC5196032

[21] Yacob N, Safii SH, Ahmad NA, et al. Denture microbiome shift and changes of salivary inflammatory markers following insertion of 3d printed removable partial pmma denture: a pilot study. BMC Oral Health. 2024;24:1216. doi: 10.1186/s12903-024-05012-z 39402561 PMC11476878

[22] Wong H-H, Hung C-H, Yip J, et al. Metagenomic characterization and comparative analysis of removable denture-wearing and non-denture-wearing individuals in healthy and diseased periodontal conditions. Microorganisms. 2024;12(6):1197. doi: 10.3390/microorganisms12061197 38930579 PMC11205920

[23] Woese CR, Fox GE. Phylogenetic structure of the prokaryotic domain: the primary kingdoms. Proc Natl Acad Sci U S A. 1977;74(11):5088–5090. doi: 10.1073/pnas.74.11.5088 270744 PMC432104

[24] Starke R, Pylro VS, Morais DK. 16S rRNA gene copy number normalization does not provide more reliable conclusions in metataxonomic surveys. Microb Ecol. 2021;81(2):535–539. doi: 10.1007/s00248-020-01586-7 32862246 PMC7835310

[25] Hu T, Chitnis N, Monos D, et al. Next-generation sequencing technologies: an overview. Hum Immunol. 2021;82(11):801–811. doi: 10.1016/j.humimm.2021.02.012 33745759

[26] Buetas E, Jordán-López M, López-Roldán A, et al. Full-Length 16S rRNA gene sequencing by pacbio improves taxonomic resolution in human microbiome samples. BMC Genomics. 2024;25:310. doi: 10.1186/s12864-024-10213-5 38528457 PMC10964587

[27] Owusu R, Savarese M. Long-read sequencing improves diagnostic rate in neuromuscular disorders. Acta Myol. 2023;42(4):123–128. doi: 10.36185/2532-1900-394 38406378 PMC10883326

[28] He Q, Kwok L-Y, Xi X, et al. The meconium microbiota shares more features with the amniotic fluid microbiota than the maternal fecal and vaginal microbiota. Gut Microbes. 2020;12(1):1794266. doi: 10.1080/19490976.2020.1794266 32744162 PMC7524391

[29] Wang Y, Zhang J, Chen X, et al. Profiling of oral microbiota in early childhood caries using single-molecule real-time sequencing. Front Microbiol. 2017;8:2244. doi: 10.3389/fmicb.2017.02244 29187843 PMC5694851

[30] Eriksson L, Lif Holgerson P, Johansson I. Saliva and tooth biofilm bacterial microbiota in adolescents in a low caries community. Sci Rep. 2017;7(1):5861. doi: 10.1038/s41598-017-06221-z 28724921 PMC5517611

[31] Li X, Yu C, Zhang B, et al. The recovery of the microbial community after plaque removal depends on periodontal health status. NPJ Biofilms Microbiomes. 2023;9(1):75. doi: 10.1038/s41522-023-00441-0 37805507 PMC10560279

[32] Wang J, Jia Z, Zhang B, et al. Tracing the accumulation of in vivo human oral microbiota elucidates microbial community dynamics at the gateway to the GI tract. Gut. 2020;69(7):1355–1356. doi: 10.1136/gutjnl-2019-318977 31227588 PMC7306975

[33] Weidlich P, Lopes de Souza MA, Oppermann RV. Evaluation of the dentogingival area during early plaque formation. J Periodontol. 2001;72(7):901–910. doi: 10.1902/jop.2001.72.7.901 11495139

[34] Weisburg WG, Barns SM, Pelletier DA, et al. 16S ribosomal DNA amplification for phylogenetic study. J Bacteriol. 1991;173(2):697–703. doi: 10.1128/jb.173.2.697-703.1991 1987160 PMC207061

[35] Callahan BJ, McMurdie PJ, Rosen MJ, et al. DADA2: high-resolution sample inference from illumina amplicon data. Nat Methods. 2016;13(7):581–583. doi: 10.1038/nmeth.3869 27214047 PMC4927377

[36] Bolyen E, Rideout JR, Dillon MR, et al. Reproducible, interactive, scalable and extensible microbiome data science using QIIME 2. Nat Biotechnol. 2019;37(8):852–857. doi: 10.1038/s41587-019-0209-9 31341288 PMC7015180

[37] Douglas GM, Maffei VJ, Zaneveld JR, et al. PICRUSt2 for prediction of metagenome functions. Nat Biotechnol. 2020;38(6):685–688. doi: 10.1038/s41587-020-0548-6 32483366 PMC7365738

[38] Schloss PD, Westcott SL, Ryabin T, et al. Introducing mothur: open-source, platform-independent, community-supported software for describing and comparing microbial communities. Appl Environ Microbiol. 2009;75(23):7537–7541. doi: 10.1128/AEM.01541-09 19801464 PMC2786419

[39] Sterzenbach T, Helbig R, Hannig C, et al. Bioadhesion in the oral cavity and approaches for biofilm management by surface modifications. Clin Oral Investig. 2020;24(12):4237–4260. doi: 10.1007/s00784-020-03646-1 PMC7666681 33111157

[40] Echhpal UR, Shah KK, Ahmed N. Effectiveness of denture cleansers on candida albicans biofilm on conventionally fabricated, computer-aided design/computer-aided manufacturing-milled, and rapid-prototyped denture base resins: an in vitro study. Cureus. 2024;16(6):e63290. doi: 10.7759/cureus.63290 39070325 PMC11283315

[41] Lim TW, Huang S, Jiang Y, et al. Characterization of pathogenic microbiome on removable prostheses with different levels of cleanliness using 2bRAD-M metagenomic sequencing. J Oral Microbiol. 2024;16(1):2317059. doi: 10.1080/20002297.2024.2317059 38410192 PMC10896157

[42] Lim TW, Huang S, Burrow MF, et al. A randomised crossover clinical trial of the efficacy of an ultrasonic cleaner combined with a denture cleanser on the microbiome on removable dentures among community-dwelling older adults. J Dent. 2025;156:105709. doi: 10.1016/j.jdent.2025.105709 40127751

[43] Karan NB, Kose R, Kalyoncu A, et al. Fatal orbital necrotizing fasciitis secondary to stenotrophomonas maltophilia associated stomatitis. J Stomatol Oral Maxillofac Surg. 2019;120(3):260–262. doi: 10.1016/j.jormas.2018.11.004 30465891

[44] Brooke JS. Stenotrophomonas maltophilia: an emerging global opportunistic pathogen. Clin Microbiol Rev. 2012;25(1):2–41. doi: 10.1128/CMR.00019-11 22232370 PMC3255966

[45] Conrads G, About I. Pathophysiology of dental caries. Monogr Oral Sci. 2018;27:1–10. doi: 10.1159/000487826 29794423

[46] Coulthwaite L, Verran J. Potential pathogenic aspects of denture plaque. Br J Biomed Sci. 2007;64(4):180–189. doi: 10.1080/09674845.2007.11732784 18236742

[47] Lourenço TGB, Heller D, Silva-Boghossian CM, et al. Microbial signature profiles of periodontally healthy and diseased patients. J Clin Periodontol. 2014;41(11):1027–1036. doi: 10.1111/jcpe.12302 25139407 PMC4213353

[48] Ng E, Tay JRH, Balan P, et al. Metagenomic sequencing provides new insights into the subgingival bacteriome and aetiopathology of periodontitis. J Periodont Res. 2021;56(2):205–218. doi: 10.1111/jre.12811 33410172

[49] Hu L, He C, Zhao C, et al. Characterization of oral candidiasis and the *Candida* species profile in patients with oral mucosal diseases. Microb Pathog. 2019;134:103575. doi: 10.1016/j.micpath.2019.103575 31175972

[50] Currie PF, Codispoti M, Mankad PS, et al. Late aortic homograft valve endocarditis caused by cardiobacterium hominis: a case report and review of the literature. Heart. 2000;83(5):579–581. doi: 10.1136/heart.83.5.579 10768915 PMC1760830

[51] Yang B, Song B, Liang J, et al. pH-Responsive DMAEM monomer for dental caries inhibition. Dent Mater. 2023;39(5):497–503. doi: 10.1016/j.dental.2023.03.019 37019743

[52] Pakroo S, Tarrah A, Takur R, et al. Limosilactobacillus fermentum ING8, a potential multifunctional non-starter strain with relevant technological properties and antimicrobial activity. Foods. 2022;11(5):703. doi: 10.3390/foods11050703 35267336 PMC8909343

[53] Chang X, Chen Y, Cui D, et al. Propionate-producing veillonella parvula regulates the malignant properties of tumor cells of OSCC. Med Oncol. 2023;40(3):98. doi: 10.1007/s12032-023-01962-6 36808012

[54] Rojas-Tapias DF, Brown EM, Temple ER, et al. Inflammation-associated nitrate facilitates ectopic colonization of oral bacterium veillonella parvula in the intestine. Nat Microbiol. 2022;7(10):1673–1685. doi: 10.1038/s41564-022-01224-7 36138166 PMC9728153

[55] Halaby MJ, McGaha TL. Amino acid transport and metabolism in myeloid function. Front Immunol. 2021;12:695238. doi: 10.3389/fimmu.2021.695238 34456909 PMC8397459

[56] Guerinot ML. Microbial iron transport. Annu Rev Microbiol. 1994;48:743–772. doi: 10.1146/annurev.mi.48.100194.003523 7826025

[57] Puri S, Kumar R, Rojas IG, et al. Iron chelator deferasirox reduces candida albicans invasion of oral epithelial cells and infection levels in murine oropharyngeal candidiasis. Antimicrob Agents Chemother. 2019;63(4):e02152-18. doi: 10.1128/AAC.02152-18 30718249 PMC6437492

[58] Alcorlo M, Abdullah MR, Steil L, et al. Molecular and structural basis of oligopeptide recognition by the ami transporter system in pneumococci. PLoS Pathog. 2024;20(6):e1011883. doi: 10.1371/journal.ppat.1011883 38838057 PMC11192437

[59] Śmiga M, Olczak T. Exploring heme and iron acquisition strategies of porphyromonas gingivalis-current facts and hypotheses. FEMS Microbiol Rev. 2025;49:fuaf019. doi: 10.1093/femsre/fuaf019 40343779 PMC12094164

[60] Akata K, Yatera K, Yamasaki K, et al. The significance of oral streptococci in patients with pneumonia with risk factors for aspiration: the bacterial floral analysis of 16S ribosomal RNA gene using bronchoalveolar lavage fluid. BMC Pulm Med. 2016;16(1):79. doi: 10.1186/s12890-016-0235-z 27169775 PMC4864928

[61] Freiria De Oliveira CA, Moraes LGDS, Vilela Teixeira AB, et al. Antimicrobial activity of cleansers on the cobalt-chromium surface of removable partial denture: a systematic review. Biofouling. 2023;39(9–10):916–927. doi: 10.1080/08927014.2023.2290120 38047547

