# Supplementary material for: Spatial and temporal dynamic changes of oral microbiome in removable partial denture wearers: a longitudinal study using full-length 16S rRNA sequencing
Source: J Oral Microbiol. 2025 Nov 26;17(1):2589655. doi: 10.1080/20002297.2025.2589655 (PMC12667312; doi:10.1080/20002297.2025.2589655)
Supplement: Supplementary material — Supplementary tables. [file ZJOM_A_2589655_SM8872.doc]

**Spatial and temporal dynamic changes of Oral Microbiome in Removable Partial Denture wearers: A Longitudinal Study Using Full-Length 16S rRNA Sequencing**

Appendix Table 1. Exclusion criteria of sample

| Exclusion Criteria |
| --- |
| <6 remain teeth per arch  Aged < 18 or women are pregnant or breastfeeding |
| Systemic diseases related to diabetes, hypertension, anaemia, xerostomia, or immunosuppression. |
| Take antibiotics/antifungal or antiviral medications, fluorides of high concentration, prebiotics or microecological regulators within 1 month |
| Have a history of smoking and drinking within 1 year, or have long-term exposure to toxic and harmful substances |
| Patients with unrepaired caries or periodontitis and have bacterial or viral infections in other areas |

Appendix Table 2. Ranking of bacterial composition at the species level by plaque collection site.

| Rank | DP | | DC | | TP | | TC | |
| --- | --- | --- | --- | --- | --- | --- | --- | --- |
|  | Species | % | Species | % | Species | % | Species | % |
| 1 | *Streptococcus_mitis* | 17.33 | *Streptococcus_mitis* | 15.12 | *Capnocytophaga_granulosa* | 9.66 | *Veillonella_parvula* | 17.20 |
| 2 | *Streptococcus_gordonii* | 13.19 | *Streptococcus_gordonii* | 14.52 | *Streptococcus_sanguinis* | 9.10 | *Streptococcus_gordonii* | 8.86 |
| 3 | *Haemophilus_parainfluenzae* | 6.58 | *Haemophilus_parainfluenzae* | 6.13 | *Veillonella_parvula* | 8.02 | *Streptococcus_sanguinis* | 8.67 |
| 4 | *Streptococcus_oralis* | 6.52 | *Streptococcus_oralis* | 6.03 | *Streptococcus_gordonii* | 6.96 | *Haemophilus_parainfluenzae* | 6.46 |
| 5 | *Veillonella_parvula* | 5.76 | *Streptococcus_parasanguinis* | 4.94 | *Streptococcus_mitis* | 4.97 | *Streptococcus_mitis* | 6.11 |
| 6 | *Streptococcus_parasanguinis* | 5.39 | *Streptococcus_sanguinis* | 4.86 | *Haemophilus_parainfluenzae* | 4.64 | *Capnocytophaga_granulosa* | 5.43 |
| 7 | *Granulicatella_adiacens* | 5.37 | *Granulicatella_adiacens* | 4.77 | *Capnocytophaga_gingivalis* | 4.18 | *Neisseria_sicca* | 3.27 |
| 8 | *Streptococcus_sanguinis* | 4.69 | *Veillonella_parvula* | 4.47 | *Neisseria_elongata* | 3.76 | *unclassified_g__Neisseria* | 2.99 |
| 9 | *Neisseria_perflava* | 3.55 | *Capnocytophaga_granulosa* | 2.45 | *Neisseria_perflava* | 3.25 | *Cardiobacterium_hominis* | 2.38 |
| 10 | *unclassified_g__Neisseria* | 2.97 | *Lautropia_mirabilis* | 2.28 | *Streptococcus_oralis* | 2.66 | *Streptococcus_oralis* | 2.32 |
| 11 | *unclassified_g__Streptococcus* | 2.43 | *unclassified_g__Streptococcus* | 2.08 | *Streptococcus_timonensis* | 2.52 | *Granulicatella_adiacens* | 2.19 |
| 12 | *Neisseria_sicca* | 2.14 | *Neisseria_sicca* | 1.98 | *unclassified_g__Neisseria* | 2.48 | *Streptococcus_timonensis* | 2.18 |
| 13 | *Streptococcus_australis* | 2.12 | *Neisseria_perflava* | 1.88 | *Streptococcus_cristatus* | 2.46 | *Capnocytophaga_gingivalis* | 1.72 |
| 14 | *Lautropia_mirabilis* | 2.10 | *Streptococcus_timonensis* | 1.86 | *Granulicatella_adiacens* | 2.18 | *unclassified_g__Streptococcus* | 1.66 |
| 15 | *Capnocytophaga_granulosa* | 1.55 | *Abiotrophia_defectiva* | 1.82 | *Ottowia_massiliensis* | 2.13 | *Neisseria_perflava* | 1.65 |
| 16 | *Streptococcus_salivarius* | 1.55 | *unclassified_g__Neisseria* | 1.70 | *Neisseria_sicca* | 1.96 | *Neisseria_elongata* | 1.59 |
| 17 | *Streptococcus_timonensis* | 1.48 | *Streptococcus_salivarius* | 1.59 | *Cardiobacterium_hominis* | 1.82 | *Ottowia_massiliensis* | 1.51 |
| 18 | *Abiotrophia_defectiva* | 1.37 | *Streptococcus_australis* | 1.58 | *Rothia_sp._CCUG_25688* | 1.59 | *Streptococcus_cristatus* | 1.47 |
| 19 | *Rothia_sp._CCUG_25688* | 0.64 | *Rothia_sp._CCUG_25688* | 1.06 | *unclassified_g__Streptococcus* | 1.55 | *Rothia_sp._CCUG_25688* | 1.09 |
| 20 | *Neisseria_elongata* | 0.44 | *Streptococcus_cristatus* | 0.58 | *Selenomonas_sp._oral_clone_EQ054* | 1.49 | *Lautropia_mirabilis* | 1.03 |
| 21 | *Streptococcus_cristatus* | 0.40 | *Neisseria_elongata* | 0.37 | *Lautropia_mirabilis* | 1.47 | *Abiotrophia_defectiva* | 0.64 |
| 22 | *Capnocytophaga_gingivalis* | 0.37 | *Capnocytophaga_gingivalis* | 0.35 | *Streptococcus_parasanguinis* | 0.80 | *Streptococcus_parasanguinis* | 0.59 |
| 23 | *Ottowia_massiliensis* | 0.23 | *Cardiobacterium_hominis* | 0.33 | *Abiotrophia_defectiva* | 0.31 | *Streptococcus_salivarius* | 0.39 |
| 24 | *Cardiobacterium_hominis* | 0.16 | *Ottowia_massiliensis* | 0.19 | *Streptococcus_australis* | 0.24 | *Selenomonas_sp._oral_clone_EQ054* | 0.23 |
| 25 | others | 11.68 | others | 17.03 | *Streptococcus_salivarius* | 0.14 | *Streptococcus_australis* | 0.20 |
| 26 |  |  |  |  | others | 19.66 | *others* | 18.18 |

Appendix Table 3. Ranking of bacterial composition at the species level of dental plaque at different times.

| Rank | t_7t | | t0t | | t1t | | t7t | | t30t | |
| --- | --- | --- | --- | --- | --- | --- | --- | --- | --- | --- |
|  | Species | % | Species | % | Species | % | Species | % | Species | % |
| 1 | *Streptococcus_sanguinis* | 9.41 | *Veillonella_parvula* | 15.69 | *Haemophilus_parainfluenzae* | 12.12 | *Veillonella_parvula* | 14.32 | *Veillonella_parvula* | 17.05 |
| 2 | *Veillonella_parvula* | 8.84 | *Streptococcus_gordonii* | 11.07 | *Streptococcus_gordonii* | 11.46 | *Capnocytophaga_granulosa* | 10.25 | *Capnocytophaga_granulosa* | 7.44 |
| 3 | *Haemophilus_parainfluenzae* | 7.19 | *Streptococcus_sanguinis* | 10.74 | *Streptococcus_sanguinis* | 10.19 | *Streptococcus_sanguinis* | 7.90 | *Streptococcus_sanguinis* | 5.96 |
| 4 | *Streptococcus_gordonii* | 6.83 | *Capnocytophaga_granulosa* | 8.64 | *Veillonella_parvula* | 9.49 | *Streptococcus_mitis* | 6.77 | *Streptococcus_mitis* | 4.99 |
| 5 | *Capnocytophaga_granulosa* | 6.58 | *unclassified_g__Neisseria* | 5.50 | *SStreptococcus_mitis* | 6.80 | *Streptococcus_gordonii* | 6.65 | *Streptococcus_gordonii* | 4.55 |
| 6 | *Streptococcus_mitis* | 4.65 | *Streptococcus_mitis* | 4.61 | *Capnocytophaga_granulosa* | 4.92 | *Capnocytophaga_gingivalis* | 4.44 | *Capnocytophaga_gingivalis* | 3.94 |
| 7 | *unclassified_g__Neisseria* | 3.31 | *Haemophilus_parainfluenzae* | 3.21 | *Neisseria_perflava* | 3.70 | *Haemophilus_parainfluenzae* | 3.38 | *Neisseria_sicca* | 3.79 |
| 8 | *Cardiobacterium_hominis* | 2.97 | *Neisseria_sicca* | 2.99 | *unclassified_g__Neisseria* | 3.57 | *Neisseria_sicca* | 3.36 | *Cardiobacterium_hominis* | 3.73 |
| 9 | *Neisseria_elongata* | 2.87 | *Streptococcus_oralis* | 2.85 | *Streptococcus_oralis* | 3.33 | *Streptococcus_timonensis* | 3.06 | *Selenomonas_sp._oral_clone_EQ054* | 2.92 |
| 10 | *Ottowia_massiliensis* | 2.85 | *Capnocytophaga_gingivalis* | 2.65 | *Neisseria_elongata* | 3.08 | *Streptococcus_cristatus* | 2.46 | *Streptococcus_cristatus* | 2.90 |
| 11 | *Rothia_sp._CCUG_25688* | 2.50 | *Neisseria_elongata* | 2.55 | *Granulicatella_adiacens* | 2.93 | *Granulicatella_adiacens* | 2.41 | *Streptococcus_oralis* | 2.73 |
| 12 | *Lautropia_mirabilis* | 2.04 | *Neisseria_perflava* | 2.49 | *Streptococcus_timonensis* | 2.10 | *Streptococcus_oralis* | 2.37 | *Campylobacter_gracilis* | 2.59 |
| 13 | *Capnocytophaga_gingivalis* | 1.96 | *Streptococcus_timonensis* | 2.07 | *unclassified_g__Streptococcus* | 1.84 | *Streptococcus_mutans* | 1.86 | *Neisseria_perflava* | 2.58 |
| 14 | *Neisseria_perflava* | 1.69 | *Cardiobacterium_hominis* | 1.63 | *Capnocytophaga_gingivalis* | 1.67 | *Neisseria_elongata* | 1.79 | *Streptococcus_timonensis* | 2.41 |
| 15 | *Granulicatella_adiacens* | 1.67 | *Streptococcus_cristatus* | 1.55 | *Neisseria_sicca* | 1.44 | *Ottowia_massiliensis* | 1.65 | *Granulicatella_adiacens* | 2.19 |
| 16 | *Streptococcus_timonensis* | 1.64 | *Granulicatella_adiacens* | 1.48 | *Streptococcus_cristatus* | 1.37 | *unclassified_g__Streptococcus* | 1.61 | *Ottowia_massiliensis* | 1.83 |
| 17 | *Streptococcus_cristatus* | 1.38 | *unclassified_g__Streptococcus* | 1.42 | *Lautropia_mirabilis* | 1.36 | *unclassified_g__Neisseria* | 1.61 | *Neisseria_elongata* | 1.65 |
| 18 | *Neisseria_sicca* | 1.37 | *Ottowia_massiliensis* | 1.20 | *Streptococcus_parasanguinis* | 0.99 | *Actinomyces_oris* | 1.61 | *Haemophilus_parainfluenzae* | 1.54 |
| 19 | *Actinomyces_oris* | 1.29 | *Rothia_sp._CCUG_25688* | 1.16 | *Cardiobacterium_hominis* | 0.97 | *Neisseria_perflava* | 1.59 | *Rothia_sp._CCUG_25688* | 1.13 |
| 20 | *Streptococcus_oralis* | 1.27 | *Lautropia_mirabilis* | 0.98 | *Rothia_sp._CCUG_25688* | 0.95 | *Cardiobacterium_hominis* | 1.57 | *unclassified_g__Streptococcus* | 1.12 |
| 21 | *Rothia_dentocariosa* | 1.20 | *Streptococcus_parasanguinis* | 0.79 | *Ottowia_massiliensis* | 0.71 | *Corynebacterium_matruchotii* | 1.40 | *Gemella_morbillorum* | 1.02 |
| 22 | *unclassified_g__Streptococcus* | 1.09 | *Capnocytophaga_sputigena* | 0.59 | *Corynebacterium_matruchotii* | 0.68 | *Lautropia_mirabilis* | 1.34 | *Streptococcus_parasanguinis* | 1.00 |
| 23 | *Cardiobacterium_sp._Marseille-Q4385* | 1.07 | *Corynebacterium_matruchotii* | 0.53 | *Capnocytophaga_sputigena* | 0.52 | *Rothia_sp._CCUG_25688* | 0.93 | *Actinomyces_oris* | 0.71 |
| 24 | *Capnocytophaga_sputigena* | 1.05 | *Rothia_dentocariosa* | 0.50 | *Actinomyces_oris* | 0.35 | *Rothia_dentocariosa* | 0.92 | *Streptococcus_mutans* | 0.59 |
| 25 | *Lautropia_dentalis* | 1.00 | *Cardiobacterium_sp._Marseille-Q4385* | 0.45 | *Rothia_dentocariosa* | 0.33 | *Streptococcus_parasanguinis* | 0.70 | *Lautropia_mirabilis* | 0.49 |
| 26 | *Corynebacterium_matruchotii* | 0.94 | *Actinomyces_oris* | 0.43 | *Gemella_morbillorum* | 0.28 | *Cardiobacterium_sp._Marseille-Q4385* | 0.65 | *unclassified_g__Neisseria* | 0.45 |
| 27 | *Selenomonas_sp._oral_clone_EQ054* | 0.93 | *Streptococcus_mutans* | 0.32 | *Streptococcus_mutans* | 0.2% | *Campylobacter_gracilis* | 0.52 | *Corynebacterium_matruchotii* | 0.37 |
| 28 | *Campylobacter_gracilis* | 0.93 | *Lautropia_dentalis* | 0.32 | *Campylobacter_gracilis* | 0.15 | *Selenomonas_sp._oral_clone_EQ054* | 0.42 | *Rothia_dentocariosa* | 0.32 |
| 29 | *Streptococcus_mutans* | 0.48 | *Selenomonas_sp._oral_clone_EQ054* | 0.26 | *Cardiobacterium_sp._Marseille-Q4385* | 0.09 | *Capnocytophaga_sputigena* | 0.36 | *Lautropia_dentalis* | 0.32 |
| 30 | *Gemella_morbillorum* | 0.19 | *Gemella_morbillorum* | 0.18 | *Lautropia_dentalis* | 0.06 | *Gemella_morbillorum* | 0.09 | *Cardiobacterium_sp._Marseille-Q4385* | 0.28 |
| 31 | *Streptococcus_parasanguinis* | 0.12 | *Campylobacter_gracilis* | 0.14 | *Selenomonas_sp._oral_clone_EQ054* | 0.04 | others | 12.04 | *Capnocytophaga_sputigena* | 0.15 |
| 32 | others | 18.66 | others | 11.00 | others | 12.29 |  |  | others | 17.22 |

Appendix Table 4. Ranking of bacterial composition at the species level RPD plaque at different times

| Rank | t1d | | t7d | | t30d | |
| --- | --- | --- | --- | --- | --- | --- |
|  | Species | % | Species | % | Species | % |
| 1 | *Streptococcus_gordonii* | 15.75 | *Streptococcus_mitis* | 18.26 | *Streptococcus_mitis* | 18.42 |
| 2 | *Streptococcus_mitis* | 7.99 | *Streptococcus_gordonii* | 13.56 | *Streptococcus_gordonii* | 15.26 |
| 3 | *Haemophilus_parainfluenzae* | 7.29 | *Streptococcus_oralis* | 8.60 | *Streptococcus_parasanguinis* | 10.53 |
| 4 | *Streptococcus_sanguinis* | 6.14 | *Haemophilus_parainfluenzae* | 7.04 | *Veillonella_parvula* | 9.06 |
| 5 | *Streptococcus_oralis* | 5.13 | *Granulicatella_adiacens* | 6.50 | *Granulicatella_adiacens* | 5.56 |
| 6 | *unclassified_g__Neisseria* | 3.55 | *Veillonella_parvula* | 5.20 | *Streptococcus_oralis* | 4.98 |
| 7 | *Lautropia_mirabilis* | 3.26 | *Streptococcus_sanguinis* | 4.74 | *Haemophilus_parainfluenzae* | 4.47 |
| 8 | *Streptococcus_parasanguinis* | 3.20 | *unclassified_g__Neisseria* | 3.60 | *Capnocytophaga_granulosa* | 4.11 |
| 9 | *Granulicatella_adiacens* | 3.17 | *Neisseria_perflava:* | 3.51 | *Streptococcus_timonensis* | 2.89 |
| 10 | *Streptococcus_salivarius* | 2.89 | *Streptococcus_parasanguinis* | 3.20 | *Streptococcus_sanguinis* | 2.76 |
| 11 | *Abiotrophia_defectiva* | 2.68 | *Neisseria_sicca* | 3.11 | *Neisseria_perflava* | 2.12 |
| 12 | *Neisseria_perflava* | 2.38 | *Streptococcus_australis* | 3.06 | *Neisseria_sicca* | 1.86 |
| 13 | *Veillonella_parvula* | 2.29 | *Lautropia_mirabilis* | 2.55 | *Streptococcus_australis* | 1.58 |
| 14 | *unclassified_g__Streptococcus* | 2.25 | *Abiotrophia_defectiva* | 1.90 | *unclassified_g__Streptococcus* | 1.32 |
| 15 | *Capnocytophaga_granulosa* | 1.90 | *Streptococcus_salivarius* | 1.16 | *Streptococcus_salivarius* | 0.84 |
| 16 | *Rothia_sp._CCUG_25688* | 1.44 | *unclassified_g__Streptococcus* | 1.07 | *Abiotrophia_defectiva* | 0.58 |
| 17 | *Klebsiella_pneumoniae* | 1.37 | *Rothia_sp._CCUG_25688* | 0.94 | *unclassified_g__Neisseria* | 0.55 |
| 18 | *Neisseria_sicca* | 1.12 | *Streptococcus_timonensis* | 0.82 | *Lautropia_mirabilis* | 0.52 |
| 19 | *Streptococcus_australis* | 0.82 | *Capnocytophaga_granulosa* | 0.52 | *Rothia_sp._CCUG_25688* | 0.23 |
| 20 | *Streptococcus_timonensis* | 0.72 | *Klebsiella_pneumoniae* | 0.23 | others | 12.36 |
| 21 | others | 24.67 | others | 10.43 |  |  |
